# Assessing the effect of genetic markers on drug immunogenicity from a mechanistic model-based approach

**DOI:** 10.1186/s12874-020-00941-z

**Published:** 2020-03-20

**Authors:** Julianne Duhazé, Miguel Caubet, Signe Hässler, Delphine Bachelet, Matthieu Allez, Florian Deisenhammer, Anna Fogdell-Hahn, Aude Gleizes, Salima Hacein-Bey-Abina, Xavier Mariette, Marc Pallardy, Philippe Broët, on behalf of the ABIRISK Consortium

**Affiliations:** 1 CHU Ste-Justine Research Center, Montréal, H3T1C5 Canada; 2 University Paris-Saclay, CESP, INSERM, Villejuif, 94807 France; 3 Immunology-Immunopathology-Immunotherapy (i3) Laboratory, UMR-S 959, INSERM, Sorbonne Université and Biotherapy Unit, Inflammation-Immunopathology-Biotherapy Department, Hôpital Pitié-Salpêtrière, AP-HP, Paris, 75000 France; 4 CESP, INSERM UMR 1018, Faculty of Medicine, Paris-Sud University, UVSQ, Paris-Saclay University, Villejuif, 94807 France; 5 Inserm CIC-EC 1425, Centre d’Investigation Clinique and Département d’Epidémiologie Biostatistique et Recherche Clinique, Hôpital Bichat, AP-HP, Paris, 75000 France; 6 Department of Gastroenterology, Hôpital Saint-Louis, AP-HP, Université Paris-Diderot, Paris, 75000 France; 7 Innsbruck Medical, Innsbruck, A-6010 - A-6080 Austria; 8 Department of Clinical Neuroscience, Karolinska Institutet, Stockholm, SE-171 76 Sweden; 9 INSERM UMR996, Faculté Pharmacie, Université Paris Sud, Châtenay-Malabry, 92290 France; 10 Clinical Immunology Laboratory, AP-HP, Le Kremlin-Bicêtre Hospital, Paris-Sud University Hospitals, Le Kremlin-Bicêtre, 94270 France; 11 UTCBS, CNRS UMR 8258, INSERM U1022, Faculty of Pharmacy, Paris-Descartes-Sorbonne-Cité University, Paris, 75000 France; 12 Centre for Immunology of Viral Infections and Autoimmune Diseases, INSERM UMR1184, AP-HP, Université Paris-Sud, Hôpitaux Universitaires Paris-Sud, Le Kremlin-Bicêtre, 94270 France; 13 Abirisk consortium WP4, Villejuif, 94807 France

**Keywords:** Genetic, Drug immunogenicity, Semi-continuous data, Two-part improper survival model, Semi-parametric

## Abstract

**Background:**

With the growth in use of biotherapic drugs in various medical fields, the occurrence of anti-drug antibodies represents nowadays a serious issue. This immune response against a drug can be due either to pre-existing antibodies or to the novel production of antibodies from B-cell clones by a fraction of the exposed subjects. Identifying genetic markers associated with the immunogenicity of biotherapeutic drugs may provide new opportunities for risk stratification before the introduction of the drug. However, real-world investigations should take into account that the population under study is a mixture of pre-immune, immune-reactive and immune-tolerant subjects.

**Method:**

In this work, we propose a novel test for assessing the effect of genetic markers on drug immunogenicity taking into account that the population under study is a mixed one. This test statistic is derived from a novel two-part semiparametric improper survival model which relies on immunological mechanistic considerations.

**Results:**

Simulation results show the good behavior of the proposed statistic as compared to a two-part logrank test. In a study on drug immunogenicity, our results highlighted findings that would have been discarded when considering classical tests.

**Conclusion:**

We propose a novel test that can be used for analyzing drug immunogenicity and is easy to implement with standard softwares. This test is also applicable for situations where one wants to test the equality of improper survival distributions of semi-continuous outcomes between two or more independent groups.

## Background

Biopharmaceuticals products (BP) such as therapeutic monoclonal antibodies are nowadays a fast-growing class of drugs whose recent use in clinic has represented a critical step forward in the treatment of many severe auto-immune diseases. Nevertheless, for some patients these BP induce an activation of the immune system, leading to the formation of antibodies against the drug. The consequences range from transient appearance of anti-drug antibodies (ADA) without any clinical significance to severe loss of efficiency by either blocking the drug or enhancing the clearance [1].

The mechanisms leading to biotherapy immunogenicity can either be patient-related (e.g: genetic background, immunological status) or treatment-related (e.g: drug characteristics and formulations) but their relative contributions to the development of ADA is currently not fully understood and still remain to be deciphered. If major achievements for minimizing product-related factors involved in immunogenicity have been recently made, thanks to the remarkable progress in biopharmaceutical engineering, there is still an urgent need for identifying non-modifiable patient-related factors that may provide a basis for stratified or personalized therapeutic approaches. However, if an extensive research has been conducted to study the immunogenic potential of the biotherapies, less has been done to identify patients who are either at high or low risk for ADA development. In this search for patient-related predictive factors of immunogenicity, the genetic diversity in immune regulatory genes, is supposed to play a major role in the development of ADA [1, 2]. If early studies about drug immunogenicity assessment have mainly relied upon response-based endpoints, time-to-event analyses are more and more often recommended for taking into account the dynamic of ADA production. For such studies, subjects that have not been previously exposed to a particular BP are followed up for a certain period of time after the first BP administration. The main outcome is the first time of ADA detection after the initial drug administration and the objective is to identify factors that are related to these time-to-events [3, 4].

The motivation behind this work is that such time-to-event analysis is not straightforward as it should take into account that the population under study is usually a mixture of pre-immune, immune-reactive and immune-tolerant subjects. Here, the so-called pre-immune subjects are those with preexisting antibodies. These preexisting ADA have been observed mainly among patients with auto-immune diseases and can originate either from the innate immune system or from the adaptive immune responses to homologous ingredients [5]. The pre-immune status of a subject can be known by doing a screening test before the first administration of the drug. In contrast, the so-called immune-tolerant subjects are those whose immune system is in a state of unresponsiveness to the exposure of the drug and thus will not produce ADA. Finally, the so-called immune-reactive subjects are those who have no pre-existing ADA and who are able to produce detectable levels of antibodies. Their time to ADA detection depends on the dynamic of the B cell clones production. However, for subjects who are not pre-immune and are censored during the study, we cannot determine their status as immune-reactive or immune-tolerant subjects.

Thus, for such analysis, we have to deal with a particular kind of semicontinous data [6, 7] with non-null proportions of zero and of infinite values coming from the pre-immune and immune-tolerant subjects, respectively. As a result, we have to use specific models that belong to the class of two-part models [6] but need to be rethought to incorporate a defective (or improper) distribution. Such defective distributions have been mainly considered in oncology for analyzing early stage of cancer. In the literature, there are two main approaches, the oldest one assumes that the population under study is composed of two subpopulations of patients: those who will not experience the event of interest and those who are likely to experience the event of interest during the follow-up. This kind of two-component mixture model incorporates the defect fraction in a parametric or semi-parametric framework (for a review see Maller and Zhou [8]). An alternative approach relies upon defining the cumulative hazard as a bounded increasing positive function [9]. This modeling which has a meaningful biological interpretation in clinical oncology belongs to the class of promotion time cure models [10].

In this work, we propose to consider a novel two-part semiparametric improper survival model that can cope with semi-continuous time to event data and from which we can derive an efficient test statistic. Here, the time-to-event is zero for pre-immune subjects with a discrete probability mass, whereas its non-zero values (immune-reactive or immune-tolerant subjects) have an improper survival distribution. This latter improper survival distribution relies upon some biological understanding of the ADA production

The paper is organized as follows. We first present the proposed two-part survival model with its interpretation. Then, we derive a general score test for the null hypothesis of a same time-to-event distribution across the different genotypes. Next, we present the results of simulation experiments comparing the behavior of our proposed score test to a two-part logrank test. Then, the clinical relevance of using the proposed test is exemplified by the analysis of the association between genetic markers and the occurrence of ADA among a cohort of treatment-naive patients suffering from auto-immune diseases and treated by biotherapies [11].

## Methods

### Modeling background

#### Notation

Let *G* denote the genotype for a biallelic marker. Here, being homozygous for the common variant is noted as *G*=[*AA*] and corresponds to the reference group. Being heterozygous and homozygous for the alternative allele are noted as *G*=[*Aa*] and *G*=[*aa*], respectively.

As the underlying genetic model is unknown, two dummy variables *G*_1_ and *G*_2_ are created for investigating various genetic models, including : dominant, additive, recessive and overdominant. In practice, *G*_1_=0,1,2 when *G*=[*AA*],[*Aa*],[*aa*] respectively and *G*_2_=1 when *G*=[*Aa*] and 0 otherwise.

Thus, if we denote *ξ*_1_ and *ξ*_2_ the effects associated with *G*_1_ and *G*_2_ respectively (see Table 1), we have a dominant genetic model when *ξ*_1_=*ξ*_2_, an additive genetic model when *ξ*_1_≠0 and *ξ*_2_=0, a recessive genetic model when *ξ*_2_=−*ξ*_1_, and an overdominant genetic model when *ξ*_1_=0 and *ξ*_2_≠0.

**Table 1:** Effects associated to a genetic variant

	**[AA]**	**[Aa]**	**[aa]**
***G***_***1***_	0	1*ξ*_1_	2*ξ*_1_
***G***_***2***_	0	1*ξ*_2_	0

*G*_1_ and *G*_2_ are dummy variables representing a genetic bi-allelic variant. [AA], [Aa] and [aa] are genotypes, with ‘A’ being the reference allele. *ξ*_1_ and *ξ*_2_ are the effects associated with *G*_1_ and *G*_2_ respectively.

In the following, we assume that the status for being a pre-immune subject can be determined before the first administration of the biotherapy. Let *Z* be the pre-immune status variable such as *Z*=1 if the subject is pre-immune and 0 otherwise.

Let denote *T* the time-to-ADA detection and *C* the censoring time. We assume that *T* and *C* satisfy the condition of independent and non informative censoring [12]. For each subject *i* (*i*=1,...*n*), *X*_*i*_=*min*(*T*_*i*_,*C*_*i*_) denotes the observed time of follow-up and $\delta _{i}=1_{(X_{i}=T_{i})}\phantom {\dot {i}\!}$ the indicator of ADA detection. We also denote $Y_{i}(t)= 1_{(t \leq X_{i})}\phantom {\dot {i}\!}$ the indicator of being at risk for the event at time *t*. Here, for *Z*=1 then *T*=0 and for *Z*=0 then *T*>0. For each patient *i*, the observed data consist of ${(X_{i},\delta _{i},Z_{i},G_{1_{i}},G_{2_{i}})}\phantom {\dot {i}\!}$.

#### Two-part improper survival model

We define the survival distribution *S*^⋆^(*t*) for these semi-continuous data such as:

*S*^⋆^(*t*;*z*)=*π*^*z*^×[(1−*π*)*S*(*t*)^1−*z*^]

where *S*(*t*) is the conditional survival function for the non-zero values of the time-to-event and *π* is the probability for the zero values. Here, the survival function *S*(*t*) is improper and its limiting value *S*(*∞*)=*a* with 0<*a*<1 is called the tail defect and represents the probability of not experiencing the event of interest.

In this work, we assume that *Z* follows a logistic regression model that depends upon the genotype. For the sake of simplicity and without loss of generality, we assume a simple additive genetic model. Thus we have:

$\pi (G_{1},G_{2})=\frac {e^{(\theta +\beta G_{1})}}{1+e^{(\theta +\beta G_{1})}}$

where *β* is the unknown regression coefficient and *θ* is the intercept.

For the reference group, $\pi (G_{[AA]}) = \pi _{0}= \frac {e^{\theta }}{1+e^{\theta }}$. When *β*=0, the proportions of pre-immune subjects are identical across the different genotypes.

For the conditional improper survival distribution *S*(*t*) we introduce a new semiparametric model which is based on immunological mechanistic considerations and is presented in the next section.

#### Conditional improper survival distribution

We propose to model the distribution of the time to ADA detection through a simplified mechanistic immunological model whereby each non pre-immune subject may or may not be able to produce ADA in response to the introduction of the biotherapy. From a biological perspective, it arises from the activation of unobservable BP-specific (T-dependent) B-cell clones that emerge and become immune-reactive ADA-producing clones. At the cellular level, each B-cell clone produces antibodies with a unique antigen-binding site with various levels of ADA affinities. Antibody affinity describes the strength of interaction between the antibody combining site and the relevant antigenic determinants in the drug. It represents an important qualitative parameter of the immune response. Positivity occurs as soon as any one of the B-cell clones is able to produce levels of ADA of sufficient amount and/or affinity for being detected by the assay.

Since B-cell clones are not directly observed, we assume a latent discrete probability distribution for the number of B-cell clones and a continuous distribution for their time-to-detection. From those latent distributions, we can deduce the marginal (or population) survival distribution of the time to ADA detection.

More precisely, let *K* (*K*=0,…,*∞*_+_) denote a latent random variable that represents the number of (unobservable) B-cell clones. Let the random variable *T*^*j*^, associated to the *j*^*th*^ latent B-cell clones, be the time-to-detection with corresponding time-to-event survival function $\phantom {\dot {i}\!}A_{j}(t)=A_{0}(t)^{U_{j}}$. Here, *A*_0_(*t*) is a baseline decreasing function such as 1≥*A*_0_(*t*)≥0 and *U*_*j*_ is a random variable that represents ADA affinity for the *j*^*th*^ clone.

Here, we suppose that *K* is distributed with probability mass function *Φ* and is supposed to be independent of *T*^*j*^. We also supposed that the *U*_*j*_ are independent and identically distributed with density function *Ψ* and independent from *K*.

For patient *i* with *k* latent B-cell clone, let denote *T*_*i*_=*min*_0≤*j*≤*k*_(*T*^*j*^) the time-to-detection of the earliest B-cell clone. The conditional (patient-specific) survival function is expressed as:

$S(t \mid K=k,(U_{1}=u_{1},...,U_{k}=u_{k}))=\Pr \left (T_{i} >t\right) =\Pr \left (T^{1}>t,\ldots,T^{k}>t\right) =A_{0}(t)^{1_{\{k \neq 0\}}\sum _{l=1}^{l=k} u_{l}}=A_{0}(t)^{1_{\{k \neq 0\}}w_{k}}$

where $w_{k}=\sum _{l=1}^{l=k} u_{l}$ is a realization of the random variable *W*_*k*_ which is the randomly stopped sum of independent random variables [13] *U*_*l*_ and 1_{*k*≠0}_=1 when *k*≠0 and 0 otherwise. This can be interpreted as a first-activation scheme model [14].

In the following, we assume a Katz distribution for the distribution of the number of latent B-cell clones (*Φ*) and a Gamma distribution *Γ*(1,*τ*) with shape parameter unity and scale parameter *τ* (*τ*≥0) for the clonal ADA affinity (*Ψ*). As a consequence, the variable *W*_*k*_ has a gamma distribution *Γ*(*k*,*τ*) (denoted in the following as *Ψ*_(*k*,*τ*)_). We also introduce *H*_0_(*t*) a positive non-decreasing function so that *A*_0_(*t*)= exp{−*H*_0_(*t*)}.

Based upon these latter assumptions, the marginal (population) survival function is given by:

$S(t)=\sum _{k=0}^{\infty _{+}} \big \{\int _{0}^{\infty _{+}} A_{0}(t)^{s}\Psi _{(k,\tau)}(s)ds \big \} \Phi (k)$

We recall that the Katz family [13] is the set of counting distributions for non-negative integers whose probability mass function satisfies the following first-order recurrence formula such as: $\Pr (x+1)=\Pr (x) \times \left (\frac {\omega +\gamma x}{1+x}\right);\text { }x=0,\ldots,\infty _{+} $ where *ω*>0 and *γ*<1. If *ω*+*γx*<0, then Pr(*x*+*j*) is equal to zero for all *j*>0. The mean and variance are $\mu = \frac {\omega }{1-\gamma }$ and $\sigma ^{2} = \frac {\omega }{(1-\gamma)^{2}}$. Moreover, it is worth noting that $\omega = \frac {\mu ^{2}}{\sigma ^{2}}$ and *γ* is linked to the dispersion index such as $\frac {\sigma ^{2}}{\mu } = (1-\gamma)^{-1}$.

The probability generating function can be written such as [13]:

$$\begin{array}{@{}rcl@{}} g(s;\mu,\gamma)&=&\left[ \left(1-\gamma \right)^{-1} \left(1-\gamma s\right) \right]^{\frac{-\omega}{\gamma} }\text{ for }\gamma \neq 0 \text{, and} \\ g(s;\omega,\gamma)&=&\exp \left[ -\omega \left(1-s\right) \right] \text{for }\gamma =0\text{ with} |s|\leq 1 \end{array} $$

This family of distribution covers a wide spectrum of discrete distributions that encompasses the binomial, the negative binomial and the Poisson distributions. If *γ*=0, we have a Poisson distribution (equidispersion) with *S*(*t*=*∞*_+_)=*e*^−*ω*^. When *γ*<0, we have underdispersion with *S*(*t*=*∞*_+_)>*e*^−*ω*^. When *γ*∈[0,1], we have overdispersion with *S*(*t*=*∞*_+_)<*e*^−*ω*^.

In the following, we assume that the distributions *Φ* and *Ψ* depend upon the genotype. In practice, we assume that for the reference group [*AA*] the latent distribution *Φ* is the Poisson distribution (*γ*=0) and *Ψ* is the Gamma distribution *Γ*(1,*τ*). In the following, the alternative genetic variant, acting in a dominant, overdominant, additive or recessive way, is associated with changes either to the distribution of the number of clones (over/underdispersion) or to the dispersion in ADA affinity (increase/decrease of *τ*).

Thus, the marginal survival function for the non pre-immune subjects depends upon the genotype such as, for the reference group:

*S*(*t*;*G*_1_=0∩*G*_2_=0)= exp{−*ω*[1−(1+*H*_0_(*t*)*τ*)^−1^]}

with *ω*>0. For the other genotypes, we have:

$${{{}\begin{aligned} &S(t;G_{1} \neq 0 \cup G_{2} \neq 0) \\&= \left[\frac{1-(\gamma_{1} G_{1} + \gamma_{2} G_{2})\left(1+H_{0}(t)\tau e^{(\alpha_{1} G_{1} + \alpha_{2} G_{2})}\right)^{-1}}{1-(\gamma_{1} G_{1} + \gamma_{2} G_{2})}\right]^{\frac{-\omega}{(\gamma_{1} G_{1} + \gamma_{2} G_{2})}} \end{aligned}}} $$

where *α*_1_,*α*_2_,*γ*_1_,*γ*_2_ are the unknown regression coefficients. The parameters *γ*_1_,*γ*_2_ are linked to the distribution of the number of the B-cell clones and *α*_1_,*α*_2_ are linked to the dispersion in ADA affinity.

When *γ*_1_=*γ*_2_=0 the improper survival distributions for each genotypes have a same defect such as *S*(*t*=*∞*_+_;*G*_1_,*G*_2_)=*e*^−*ω*^. The biological interpretation is that the alternative allele has no effect on the proportion of immune-tolerant. When *γ*_1_≠0 or *γ*_2_≠0, the improper survival distribution varies between genotypes. The biological interpretation is that the alternative allele is associated with changes in the distribution of the number of clones and consequently with changes in the proportion of immune-tolerant.

We write the improper survival model in terms of the hazard functions and take its first-order Taylor expansion of *γ*_1_=*γ*_2_=0.

$$\begin{array}{*{20}l} \lambda(t \mid G_{1},G_{2})&=k_{0}(t)\times e^{\alpha G-2\log \left[1+H_{0}(t)\tau e^{\alpha G}\right]} \\ &{\kern5pt}{+\gamma G \left[1 + H_{0}(t)\tau e^{\alpha G}\right]^{-1}} =k_{0}(t)e^{\Upsilon(t)} \end{array} $$

where *αG*=*α*_1_*G*_1_+*α*_2_*G*_2_, *γG*=*γ*_1_*G*_1_+*γ*_2_*G*_2_, *k*_0_(*t*)=*ωτh*_0_(*t*) is a baseline hazard function with $h_{0}(t)=\frac {\partial H_{0}(t)}{\partial t}$ and *Υ*(*t*) is a function of *γ*_1_;*γ*_2_;*α*_1_;*α*_2_;*G*_1_;*G*_2_. This multiplicative hazard formulation will be used in the following.

### The proposed statistic

In this work, the general null hypothesis to be tested is defined by *H*_0_:*β*=*γ*_1_=*γ*_2_=*α*_1_=*α*_2_=0 and corresponds to a same survival distribution across genotypes against differences that can be associated to changes linked either to the pre-immune fraction (*β*≠0) or to the improper survival distribution for the non pre-immune subjects. From our mechanistic model, these differences are related either to the dispersion of the latent distribution for the B-cell clones (*γ*_1_≠0 or *γ*_2_≠0) or to the ADA affinity (*α*_1_≠0 or *α*_2_≠0).

In the following, we derive under the null hypothesis a score statistic for testing *H*_0_.

The log-likelihood $LL_{0}\left (\beta,\gamma _{1},\gamma _{2},\alpha _{1},\alpha _{2};G_{1_{i}},G_{2_{i}},Z_{i}\right)$ derived under the working model can be expressed as the sum of two terms *LL*_0_=*LL*_1_+*LL*_2_ where the first term involves only the parameter *β* whereas the second term involves the parameters *γ*_1_,*γ*_2_,*α*_1_;*α*_2_.

$LL_{1} = \sum _{i=1}^{n}Z_{i}\left \{\theta +\beta G_{1_{i}}-log\left (1+e^{(\theta +\beta G_{1_{i}})}\right)\right \} + (1-Z_{i}) \left \{-log\left (1+e^{(\theta +\beta G_{1_{i}})}\right)\right \}$

$LL_{2}= \sum _{i=1}^{n} (1-Z_{i})\delta _{i} \left [log(k_{0}(t))+\Upsilon (t)\right ] - (1-Z_{i}) \int _{0}^{\infty } k_{0}(s) e^{\Upsilon (s)}ds$

If we consider the non-parametric form for *k*_0_(*t*) that puts point mass *k*_0*j*_ at the *J* ordered failure times *t*_(*j*)_, then $K_{0}(t)=\sum _{j=1}^{J} k_{0j}(t_{(j)} \leq t)$, as our improper survival model is a multiplicative model, the profile likelihood (LL2) (profile out *K*_0_) leads to the classical log partial likelihood function from which a score test can be derived under the null hypothesis [15, 16]. Here the log-partial likelihood is: $LPL_{2}= \sum _{i=1}^{n} (1-Z_{i}) \delta _{i} \Upsilon (t_{i}) - \sum _{j=1}^{n}Y_{j}(t_{i}) (1-Z_{i})e^{\Upsilon (t_{i})}$.

Thus, one can obtain two score statistics by separately computing the components of the score statistics derived from the first derivatives of *LL*_1_ and the log partial likelihood *LPL*_2_ with respect to *β* and *α*_1_,*α*_2_,*γ*_1_,*γ*_2_ evaluated under *H*_0_, respectively.

The first score statistic is as follows:

$\hat {V}_{H_{0},\beta }=\frac {\partial LL_{1}}{\partial \beta } = \sum \limits _{i=1}^{n}G_{1_{i}}\left \{Z_{i}-\pi _{0} \right \}$

with $\pi _{0}=\frac {e^{\theta }}{1+e^{\theta }}$ the probability of being a pre-immune under the null hypothesis *H*_0_.

The components of the second score statistic are such as:

$$\begin{array}{@{}rcl@{}} \hat{U}_{H_{0},\alpha_{1}}=\frac{\partial LPL_{2}}{\partial \alpha_{1}} &=&\sum\limits_{i=1}^{n}\delta_{i}W_{1}(t)\left\{ G_{1_{i}}- \frac{\sum\limits_{k=1}^{n}Y_{k}\left(t_{i}\right) G_{1_{k}}}{ \sum\limits_{k=1}^{n}Y_{k}\left(t_{i}\right) }\right\} \\ \hat{U}_{H_{0},\alpha_{2}}=\frac{\partial LPL_{2}}{\partial \alpha_{2}} &=&\sum\limits_{i=1}^{n}\delta_{i}W_{1}(t)\left\{ G_{2_{i}}- \frac{\sum\limits_{k=1}^{n}Y_{k}\left(t_{i}\right) G_{2_{k}}}{ \sum\limits_{k=1}^{n}Y_{k}\left((t_{i}\right) }\right\} \\ \hat{U}_{H_{0},\gamma_{1}}=\frac{\partial LPL_{2}}{\partial \gamma_{1}} &=&\sum\limits_{i=1}^{n}\delta_{i}W_{2}(t)\left\{ G_{1_{i}}-\frac{ \sum\limits_{k=1}^{n}Y_{k}\left(t_{i}\right) G_{1_{k}}}{\sum \limits_{k=1}^{n}Y_{k}\left(t_{i}\right) }\right\} \\ \hat{U}_{H_{0},\gamma_{2}}=\frac{\partial LPL_{2}}{\partial \gamma_{2}} &=&\sum\limits_{i=1}^{n}\delta_{i}W_{2}(t)\left\{ G_{2_{i}}-\frac{ \sum\limits_{k=1}^{n}Y_{k}\left(t_{i}\right) G_{2_{k}}}{\sum \limits_{k=1}^{n}Y_{k}\left(t_{i}\right) }\right\} \end{array} $$

where $\phantom {\dot {i}\!}W_{1}(t)=1-2\big (\frac {\Lambda (t)}{\omega }\big)$ and $\phantom {\dot {i}\!}W_{2}(t)=1-\big (\frac {\Lambda (t)}{\omega }\big)$ with *Λ*(*t*) as the bounded cumulative hazard function under the null hypothesis *H*_0_.

For computing these two score statistics, we should substitute *Λ*(*t*), *ω* and *π*_0_ by efficient estimators computed under the null hypothesis. Here $\hat {\pi _{0}}$ is the observed proportion of pre-immune subjects computed under the null hypothesis. The quantity $\phantom {\dot {i}\!}\hat {\Lambda }(t)=\sum _{i=1}^{n}\int _{0}^{t}\{\sum _{k=1}^{n}Y_{k}(s)\}^{-1}dN_{i}(s)$, where $\phantom {\dot {i}\!}N_{i}(t)=1_{\{X_{i}\leq t,\delta _{i}=1\}}$ is the left-continuous version of the Nelson-Aalen estimator for the cumulative hazard [15, 17] obtained by using the pooled sample, and $\hat {\omega }=\hat {\Lambda }(t_{\max })$ is the maximum value of this estimator computed at the last observed failure time *t*_max_. Here, $\hat {\omega }$ is an efficient estimator of *ω* since we assume that the immune-reactive patients should experience the event within the one-year follow-up.

For the two scores, their corresponding information scalar and matrix, respectively $\hat {J}_{H_{0}}$ and $\hat {I}_{H_{0}}$, are obtained as presented in the Supplementary material. They are are computed by using efficient estimators of *W*_1_(*t*) and *W*_2_(*t*) as given above. Then,

$S_{1}=\hat {V}_{H_{0}}{\hat {J}_{H_{0}}}^{-1}\hat {V}_{H_{0}}$ and $S_{2}=\left (\hat {U}_{H_{0},\alpha _{1}},\hat {U}_{H_{0},\alpha _{2}},\hat {U}_{H_{0},\gamma _{1}},\hat {U}_{H_{0},\gamma _{2}}\right) \hat {I}_{H_{0}}^{-1}\\ \left (\hat {U}_{H_{0},\alpha _{1}},\hat {U}_{H_{0},\alpha _{2}},\hat {U}_{H_{0},\gamma _{1}},\hat {U}_{H_{0},\gamma _{2}}\right)^{^{\prime }}$.

Thus, the statistic : $S_{H_{0}}=S_{1}+S_{2}$ is asymptotically distributed under *H*_0_ as a chi-square with five degrees of freedom. This test statistic is referred as TWIST, for TWo-part Improper Survival Test in the following

### Statistical test comparison

We compared our proposed test to a Two-part LogRank Test, hereafter referred as TLRT. Here, the TLRT is derived in the same spirit as proposed by Lachenbruch (2001)[7] where the first part is the score test under the logistic model and the second part is a k-sample logrank test. Here, *k*=3 for the three genotype groups.

## Results

### Data generation

Monte Carlo simulations were performed in order to evaluate the behavior of the TWIST. We reported the size of the proposed test, as well as its power properties with those obtained with a TLRT. Individual’s genotype information was generated by summing the values of two Bernoulli variables independently drawn, with mean: 0.1, 0.2 and 0.3. This value corresponds to a pseudo-minor allele frequency (MAF), that is to say the proportion of [*a*] allele in the simulated population.

The status for being pre-immune was sampled from a Bernoulli variable, with mean of 0.05, 0.1 or 0.2. The values for *β* are chosen so that the pre-immune fractions are equal across the different groups (then *β*=0), or different according to the group ($\beta = log(2), log(\frac {1}{2})$).

For the non pre-immune and to evaluate the impact of departures from the reference latent distribution, we have considered scenarii with equi, under or overdispersed latent distributions corresponding to Poisson, Binomial and Negative binomial distributions, respectively.

Data were generated such as, for the reference group with [*AA*] genotype, *S*_*AA*_(*t*)= exp[−*ω*(1−(1+*t*)^−1^)] and for the two other genotypes [*aa*] and [*Aa*], $S(t) = \left [\frac {1-(\gamma _{1} G_{1} + \gamma _{2} G_{2})\left (1+ t e^{\alpha _{1} G_{1} + \alpha _{2} G_{2}}\right)^{-1}}{1-(\gamma _{1} G_{1} + \gamma _{2} G_{2})}\right ]^{\frac {-\omega }{\gamma _{1} G_{1} + \gamma _{2} G_{2}}}$.

The tail defect for the reference group was chosen such as: *S*_*AA*_(*∞*_+_)=*e*^−*ω*^=0.50. We also investigated the case where *S*_*AA*_(*∞*_+_)=*e*^−*ω*^=0.30.

The values for *γ*_1_ and *γ*_2_ were chosen accordingly to have over, equi or underdispersion, under an additive, dominant, recessive or overdominant genetic model. The values for *α*_1_ and *α*_2_ were chosen in such a way as to the risk $e^{\alpha _{1} G_{1} + \alpha _{2} G_{2}} = 2\phantom {\dot {i}\!}$. Thus, when the genetic model is dominant, *α*_1_=0.5×*log*(2) and *α*_2_=0.5×*log*(2); when the genetic model is additive, *α*_1_=0.5×*log*(2) and *α*_2_=0; when the genetic model is recessive, *α*_1_=0.5×*log*(2) and *α*_2_=−0.5×*log*(2) and when the genetic model is overdominant, *α*_1_=0 and *α*_2_=*log*(2).

According to the model, *α* and *γ* have the same directional effects when they are of opposite signs. Thereby, when *γ*<0 and *e*^*α*^>1 there is a global underdispersion, and the reverse for *γ*∈]0−1] and *e*^*α*^<1.

The censoring time *C* was generated from a uniform distribution with parameter value computed from the chosen percentage of censoring. The percentage of censoring refers only to the percentage of censored observations without the tail defect fraction. We investigated no censoring and 20% censoring.

The total number of subjects was chosen to be 1,000 and for each configuration of parameters, 1,000 replications were performed. The levels and powers of the TWIST and the TLRT were estimated with a 0.05 significance level.

### Simulation results

The estimated level of the TWIST under its proper null hypothesis fell within the binomial range [0.036−0.064] (see the underlined values in Table 2b and in Tables S1 to S6 in the Supplementary material).

**Table 2:** Power of the TWIST compared to the TLRT when *α* and *γ* are additive

	**Underdispersion**		**Equidispersion**		**Overdispersion**	
	***(γ***_***1***_***=−0.35*and*γ***_***2***_***=0)***		***(γ***_***1***_***=0*and*γ***_***2***_***=0)***		***(γ***_***1***_***=0.10*and*γ***_***2***_***=0)***	
***e***^***β***^	**TWIST**	**TLRT**	**TWIST**	**TLRT**	**TWIST**	**TLRT**
(a) *α* has an additive effect and *e*^*α*^>1						
**2**	0.95	0.56	0.42	0.08	0.49	0.42
**1**	0.93	0.36	0.29	0.02	0.37	0.28
**0.50**	0.95	0.47	0.32	0.03	0.42	0.32
(b) *α* has no effect, *e*^*α*^=1						
**2**	0.50	0.44	0.09	0.10	0.59	0.60
**1**	0.36	0.25	0.04	0.02	0.50	0.48
**0.50**	0.45	0.34	0.04	0.04	0.53	0.53
(c) *α* has an additive effect and *e*^*α*^<1						
**2**	0.22	0.32	0.41	0.12	0.95	0.77
**1**	0.11	0.14	0.30	0.04	0.91	0.65
**0.50**	0.14	0.18	0.35	0.05	0.96	0.74

(b) The underlined values show the estimated level of the type I error.

(c) The tail defects for the [AA], [Aa] and [aa] groups are respectively : 0.50, 0.55 and 0.59 for *γ*_1_= -0.35, and 0.50, 0.44 and 0.37 for *γ*_1_= 0.10. The proportion of pre-immune subjects is 10% and the MAF is 20%.

Just below, we comment the results of Table 2. It displays the estimated power gains in the TWIST and the TLRT for different values of the parameters *α*, *β* and *γ*, for a 10% proportion of pre-immune subjects and a minor allele frequency of 20%. In Table 2, both *α* and *γ* have additive effects, hence, *α*_2_=0 and *γ*_2_=0. The results for other genetic models are available in the Supplementary material.

We first comment the configurations where *α* has no effect (*e*^*α*^=1, Table 2b). In this case, the TWIST’s power is higher than the TLRT’s when *γ*_1_=−0.35 (underdispersion). The TWIST’s power is very close to the TLRT’s when *γ*_1_=0 or *γ*_1_=0.10. In these cases, the difference between the TWIST’s power and the TLRT’s vary from 0 to 0.02. When only *β* has an effect, the TWIST has a very low power. For example, when *e*^*β*^=0.5, the TWIST’s power is 0.04.

We will now comment the configurations where *α* has a non-null effect (Table 2a and c).

When parameters *α* and *γ* have the same directional effect, the TWIST’s power is always higher than the TLRT’s, with a difference varying from 0.18 to 0.57. As an example, when both *α* and *γ* increase the risk and *e*^*β*^=2, the TWIST’s and TLRT’s powers are 0.95 and 0.56 respectively.

When *γ* has no effect, the TWIST’s power is higher than the TLRT’s, with a difference varying from 0.26 to 0.34.

When *α* and *γ* have opposite directional effect, the TWIST’s power values are close to the TLRT’s. The difference between the powers of the two tests varies from −0.10 to 0.10. As an example, when *γ*_1_=0.10, *e*^*α*^>1 and *e*^*β*^=2, the TWIST’s and TLRT’s powers are 0.49 and 0.42 respectively.

As expected, when *e*^*β*^=1, the power of the TWIST is inferior to the one obtained with *e*^*β*^≠1.

The results of simulations with *α* and *γ* acting under a same genetic model other than additive are not displayed in this section but are joined in the Supplementarymaterial (Table S6). The TWIST’s power values obtained with *α* and *γ* both dominant, overdominant or recessive are different from what we described above but follow the same global trends. Moreover, according to the genetic model, the power values are different. When both *α* and *γ* are dominant or overdominant, the TWIST’s power values are higher than the ones obtained under an additive genetic model. When both *α* and *γ* are recessive, the TWIST’s power values are lower than what is obtained under an additive genetic model.

We performed simulations with the same parameters as described above but with 20% censoring (see Table S1 in the Supplementary material). As expected, the TWIST’s power values when there is 20% censoring are lower than when there is no censoring. Moreover, the difference between the TWIST’s power and the TLRT’s is lower with 20% censoring compared to no censoring.

Tables S2 to S6 in the Supplementary material display the results of simulations performed with all parameters equal to those of Table 2, unless otherwise specified. For all these configurations, the trends are similar to what we detailed above about Table 2, with changes in power values and gains. We describe briefly just below the results displayed in those additional tables.

With a minor allele frequency of 10%, as expected, the TWIST’s power is lower than when the MAF is 20%, and with a 30% MAF, it is higher (see Tables S4 and S5).

With a proportion of pre-immune subjects in the reference group of 5% or 20%, the TWIST’s power is similar to what we obtain when this proportion is 10%, with slight variations according to the configuration of the parameters (see Additional file 1: Tables S4 and S5).

When the tail defect is 30%, the TWIST’s power is higher than when the tail defect is 50% (see Table S6).

We also performed simulations with *α* having a different genetic model than *γ*, those results are displayed in Tables S7 and S8 of the Supplementary material and display similar trends than what is described above, with different power values according to the genetic model.

### Biotherapeutic immunogenicity study

We used our novel test to look for association between genetic variants and occurrence of ADA. The population study consists of 469 patients from the ABIRISK consortium cohort [11] suffering from auto-immune diseases and treated by biotherapies. These patients were naive for the biotherapies before the study and were followed during twelve months. The outcome was the time between the date of the first dose of biotherapy and the first detection of anti-drug antibodies (ADA). Patients without ADA occurrence were censored at the date of their last clinic visit. Among the 469 subjects, 17 (3.6*%*) had pre-existing ADA and 129 (27.5*%*) developed ADA during the one-year follow-up.

Exploratory analyses were performed on a list of 1,697 genes linked to immunity. We selected the genetic variants with a minor allele frequency greater than 0.15, for a total of 19,745 variants. At a 10% false discovery rate level [18], five genetic variants were significantly associated with ADA occurrence with the TWIST, and none with the TLRT. Among these five variants, we highlight one from the fibroblast growth factor signaling pathway which test statistic is 28.96 (*q-value* =0.093) with the TWIST and 21.87 with the TLRT (*q-value* =0.34). Taken separately, the first and second components of the TWIST statistic have values of 8.02 and 20.94 respectively. It’s worth noting that if we restricted the analyses to the 452 non pre-immune subjects with the same 10% false discovery rate, this genetic variant was not selected (*q-value* =0.39).

**Fig. 1:**
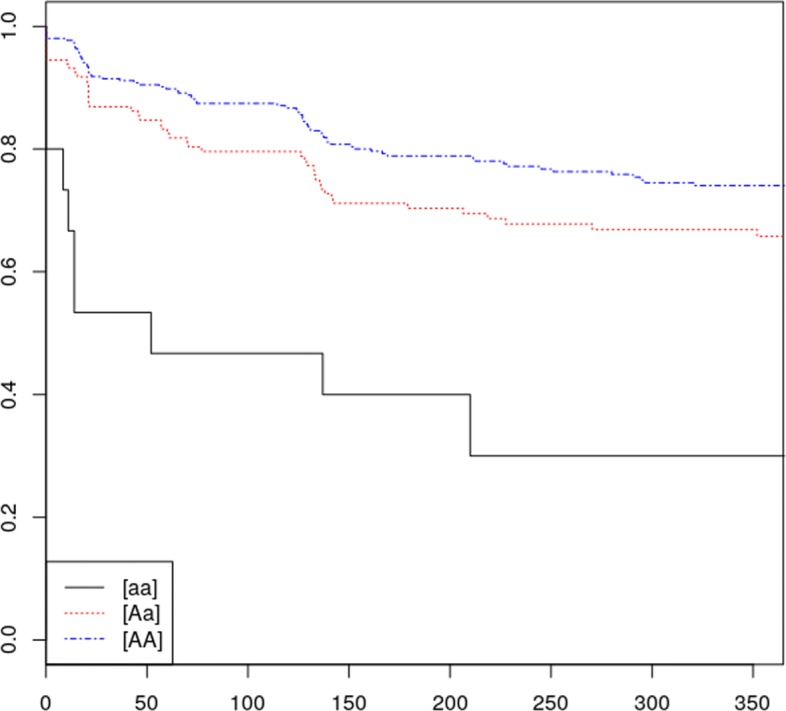
Survival curve of the fibroblast growth factor signaling pathway variant. We named ‘A’ the reference allele and ‘a’ the alternative allele

This latter finding emphasizes the use of the TWIST and the inclusion of the pre-immune subjects for the analyses. Figure 1 shows the survival curves for the three genotypic groups of the highlighted variant. We found that the rare variant is associated with a higher occurrence of ADA in both pre-immune and non pre-immune subjects.

## Discussion

In this paper, we proposed a test for assessing the effect of genetic markers on drug imunogenicity. This test is based on a score statistic derived from a two-part semi-parametric improper survival model which allows to test the effect of genetic markers on the time-to-detection of ADA. It extends classical two-part models for semi-continuous data to the case where we can expect a mass at infinity. This model has a mechanistic interpretation of the ADA production by the B-cell clones. It allows different types of departures from equality of the two-part improper survival distributions. The biological interpretation of these departures are linked either to the proportion of pre-immune subjects or to the dynamic of ADA production. In this latter case, genetic variants can be associated with changes in the number of clones or to their ADA affinities. As seen from our simulations, under the null hypothesis, our proposed test maintains a correct type I error in all the investigated configurations. Looking at the simulation results under the alternative hypotheses, we see that power gains of the proposed test are better than that of the two-part logrank test in most situations. In particular, we see that striking gains in power can be achieved by the TWIST as compared to the two-part logrank test when the parameters have the same directional effect, which is the case that we could expect the most in applications on real data. Since for complex diseases the underlying model is unknown, we have not restricted our test to one of the common genetic models. For various combinations of the classical genetic models, we obtain good performances with the proposed test. Moreover, it is worth noting that a straightforward extension of this test statistic can be done to investigate specific genetic models. Power gains decrease when the plateau or the percentage of censoring increase, which is not surprising since there are less events. Likewise, when the groups are too unbalanced, for example when the minor allele frequency is low or when the underlying genetic model is recessive, power gains decrease. The simulation results also show that our test performs well when there is a non negligible proportion of pre-immune subjects.

Notwithstanding the good behavior of the proposed test, some limitations should be mentioned. In this work, we have considered a proportional effect of the studied variable on the time-to-detection associated with the B-cell clones. However, if this hypothesis is not true (e.g. in case of accelerated failure time), the power of the test will be reduced. Here, we have considered a Katz family for the distribution of the number of latent B-cell clones. This modelisation provides a flexible probabilistic modeling approach but at the expense of increasing the number of parameters to be tested. Moreover, it is worth keeping in mind that this test performs best in situations where a sufficient length of follow-up is provided. It means that we need a window of observation long enough for the last potential event among immune reactive to occur within it. In other words, we should provide a follow-up adequate for detecting the presence of immune-tolerant subjects in the study population. If this condition is unmet, the power of the test is reduced.

We used the proposed test for analyzing the relationship between some genetics loci and drug immunogenicity among a cohort of patient with auto-immune diseases and naive for the tested drugs. In this study, 3.6% of the patients were pre-immune subjects. We found that the alternative alleles of five variants on immunity genes are significantly associated with an increased risk of developing ADA. The result for one variant from the fibroblast growth factor signaling pathway is interesting since it has an effect on both the fraction of pre-immune subjects and the time of ADA occurrence. For this case, the inclusion of the pre-immune subjects in the analyses allows to detect the association of the variant with ADA occurrence.

Thus, we think that our test provides a new way of assessing the effect of genetic markers on drug immunogenicity, while incorporating some biological understanding of the ADA production. Our model-based approach can be considered as a convenient representation of complex patterns of genetic effects which allows detection of departure from the null hypothesis model.

It is worth noting that since the proposed test statistic gives equal weights to its two parts, its global power can be reduced when one of them has a smaller effect size (e.g. pre-immune subjects). Following the idea introduced in the paper of Hu and Proschan [19], it could be interesting to consider a linear combination of the two components of our proposed test statistic. As in the work of Hu and Proschan, we could assign a lesser weight to the component with the smaller a priori effect than to the other. However, it requires additional work for deriving the probability density function of the optimal linear combination of the two components of the test statistic. Moreover, further works should also be done for deriving simple test statistics for testing separately each component for the non pre-immune subjects.

In view of the gains in power obtained by the proposed test, its use can be recommended in many clinical situations where unwanted drug immunogenicity occurs such as in oncology and clinical immunology. It can also be used to evaluate response to vaccination for populations where pre-immune and non pre-immune patients exist. Moreover, the proposed test can also be extended to take other factors into account such as different treatments, by developing a stratified version with strata defined by the levels of the factors.

It can also be used to evaluate response to vaccination for populations where pre-immune and non pre-immune patients exist. Moreover, the proposed test can also be extended to take other factors into account such as different treatments, by developing a stratified version with strata defined by the levels of the factors.

## Conclusion

In this study, we proposed a novel test statistic for assessing the effect of genetic markers on drug immunogenicity taking into account that the population under study is a mixed one. This test statistic, which is easy to implement with standard softwares, is also applicable in situations where one wants to test the equality of improper survival distributions of semi-continuous outcomes between two or more independent groups.

## Supplementary information

**Additional file 1** Supplementary material.

## Data Availability

The data analyzed in this study were collected in the context of the ABIRISK project by ABIRISK partners. Access to the minimal dataset underlying the findings can be obtained by interested researchers upon request to the ABIRISK Sustainability Scientific Committee by submission of an analysis plan. The analysis plan should explain the purpose of the use of the data and confirm the intention to use the data only for replication studies concerning anti-drug inhibitors, since this is the limitation of the ethical permission on how this data can be used. The contact person of the ABIRISK Sustainability Scientific Committee to whom the requests should be sent is Marc Pallardy (marc.pallardy@inserm.fr).

## Abbreviations

ADA: Anti-drug antibodies
BP: Biopharmaceutical products
MAF: Minor allele frequency
TLRT: Two-part logrank test
TWIST: Two-part improper survival test
