# Supplementary material for: Assessing the effect of genetic markers on drug immunogenicity from a mechanistic model-based approach
Source: BMC Med Res Methodol. 2020 Mar 20;20:69. doi: 10.1186/s12874-020-00941-z (PMC7082907; doi:10.1186/s12874-020-00941-z)
Supplement: Supplementary file 1 — Additional file 1 Supplementary material. [file 12874_2020_941_MOESM1_ESM.pdf]

Additional Files

Information scalar and matrix for the test statistic

The elements of the information matrices are computed under the null hypothesis by substituting π_0, W_1 and W_2 by their efficient estimators.

Second derivative of $LL1$ for the information scalar $\hat{J}_{H_0}$:

$$\hat{J}_{H_0} = \frac{\partial^2 LL_1}{\partial^2 \beta} \quad = \quad \sum_{i=1}^{n} G_{1_i}^2 \{Z_i - \pi_0\}$$

Second derivatives of $LL2$ for the information matrix $\hat{I}_{H_0}$:

$$\hat{\mathcal{V}}_{H_0,\alpha_1} = \frac{\partial^2 LPL_2}{\partial^2 \alpha_1} \quad = \quad \sum_{i=1}^{n} \delta_i W_1(t)^2 \left\{ \frac{\sum_{k=1}^{n} Y_k(t_i) G_{1_k}^2 \sum_{k=1}^{n} Y_k(t_i) - \left(\sum_{k=1}^{n} Y_k(t_i) G_{1_k} \right)^2}{\left(\sum_{k=1}^{n} Y_k(t_i) \right)^2} \right\}$$

$$\hat{\mathcal{V}}_{H_0,\alpha_2} = \frac{\partial^2 LPL_2}{\partial^2 \alpha_2} \quad = \quad \sum_{i=1}^{n} \delta_i W_1(t)^2 \left\{ \frac{\sum_{k=1}^{n} Y_k(t_i) G_{2_k}^2 \sum_{k=1}^{n} Y_k(t_i) - \left(\sum_{k=1}^{n} Y_k(t_i) G_{2_k} \right)^2}{\left(\sum_{k=1}^{n} Y_k(t_i) \right)^2} \right\}$$

$$\hat{\mathcal{V}}_{H_0,\alpha_1\alpha_2} = \frac{\partial^2 LPL_2}{\partial \alpha_1 \partial \alpha_2} \quad = \quad \sum_{i=1}^{n} \delta_i W_1(t)^2 \left\{ \frac{\sum_{k=1}^{n} Y_k(t_i) G_{1_k} G_{2_k} \sum_{k=1}^{n} Y_k(t_i) - \sum_{k=1}^{n} Y_k(t_i) G_{1_k} \sum_{k=1}^{n} Y_k(t_i) G_{2_k}}{\left(\sum_{k=1}^{n} Y_k(t_i) \right)^2} \right\}$$

$$\hat{\mathcal{V}}_{H_0,\gamma_1} = \frac{\partial^2 LPL_2}{\partial^2 \gamma_1} \quad = \quad \sum_{i=1}^{n} \delta_i W_2(t)^2 \left\{ \frac{\sum_{k=1}^{n} Y_k(t_i) G_{1_k}^2 \sum_{k=1}^{n} Y_k(t_i) - \left(\sum_{k=1}^{n} Y_k(t_i) G_{1_k} \right)^2}{\left(\sum_{k=1}^{n} Y_k(t_i) \right)^2} \right\}$$

$$\hat{\mathcal{V}}_{H_0,\gamma_2} = \frac{\partial^2 LPL_2}{\partial^2 \gamma_2} \quad = \quad \sum_{i=1}^{n} \delta_i W_2(t)^2 \left\{ \frac{\sum_{k=1}^{n} Y_k(t_i) G_{2_k}^2 \sum_{k=1}^{n} Y_k(t_i) - \left(\sum_{k=1}^{n} Y_k(t_i) G_{2_k} \right)^2}{\left(\sum_{k=1}^{n} Y_k(t_i) \right)^2} \right\}$$

$$\hat{\mathcal{V}}_{H_0,\gamma_1\gamma_2} = \frac{\partial^2 LPL_2}{\partial \gamma_1 \partial \gamma_2} \quad = \quad \sum_{i=1}^{n} \delta_i W_2(t)^2 \left\{ \frac{\sum_{k=1}^{n} Y_k(t_i) G_{1_k} G_{2_k} \sum_{k=1}^{n} Y_k(t_i) - \sum_{k=1}^{n} Y_k(t_i) G_{1_k} \sum_{k=1}^{n} Y_k(t_i) G_{2_k}}{\left(\sum_{k=1}^{n} Y_k(t_i) \right)^2} \right\}$$

$$\hat{\mathcal{V}}_{H_0,\alpha_1\gamma_1} = \frac{\partial^2 LPL_2}{\partial \alpha_1 \partial \gamma_1} \quad = \quad \sum_{i=1}^{n} \delta_i W_2(t) W_1(t) \left\{ \frac{\sum_{k=1}^{n} Y_k(t_i) G_{1_k}^2 \sum_{k=1}^{n} Y_k(t_i) - \left(\sum_{k=1}^{n} Y_k(t_i) G_{1_k} \right)^2}{\left(\sum_{k=1}^{n} Y_k(t_i) \right)^2} \right\}$$

$$\hat{\mathcal{V}}_{H_0,\alpha_1\gamma_2} = \frac{\partial^2 LPL_2}{\partial \alpha_1 \partial \gamma_2} \quad = \quad \sum_{i=1}^{n} \delta_i W_2(t) W_1(t) \left\{ \frac{\sum_{k=1}^{n} Y_k(t_i) G_{1_k} G_{2_k} \sum_{k=1}^{n} Y_k(t_i) - \sum_{k=1}^{n} Y_k(t_i) G_{1_k} \sum_{k=1}^{n} Y_k(t_i) G_{2_k}}{\left(\sum_{k=1}^{n} Y_k(t_i) \right)^2} \right\}$$

$$\hat{\mathcal{V}}_{H_0,\alpha_2\gamma_1} = \frac{\partial^2 LPL_2}{\partial \alpha_2 \partial \gamma_1} \quad = \quad \sum_{i=1}^{n} \delta_i W_2(t) W_1(t) \left\{ \frac{\sum_{k=1}^{n} Y_k(t_i) G_{1_k} G_{2_k} \sum_{k=1}^{n} Y_k(t_i) - \sum_{k=1}^{n} Y_k(t_i) G_{1_k} \sum_{k=1}^{n} Y_k(t_i) G_{2_k}}{\left(\sum_{k=1}^{n} Y_k(t_i) \right)^2} \right\}$$

$$\hat{\mathcal{V}}_{H_0,\alpha_2\gamma_2} = \frac{\partial^2 LPL_2}{\partial \alpha_2 \partial \gamma_2} \quad = \quad \sum_{i=1}^{n} \delta_i W_2(t) W_1(t) \left\{ \frac{\sum_{k=1}^{n} Y_k(t_i) G_{2_k}^2 \sum_{k=1}^{n} Y_k(t_i) - \left(\sum_{k=1}^{n} Y_k(t_i) G_{2_k} \right)^2}{\left(\sum_{k=1}^{n} Y_k(t_i) \right)^2} \right\}$$

and

$$\hat{I}_{H_0} = \begin{pmatrix} \hat{\mathcal{V}}_{H_0,\alpha_1} \hat{\mathcal{V}}_{H_0,\alpha_1\alpha_2} \hat{\mathcal{V}}_{H_0,\alpha_1\gamma_1} \hat{\mathcal{V}}_{H_0,\alpha_1\gamma_2} \\ \hat{\mathcal{V}}_{H_0,\alpha_1\alpha_2} \hat{\mathcal{V}}_{H_0,\alpha_2} \hat{\mathcal{V}}_{H_0,\alpha_2\gamma_1} \hat{\mathcal{V}}_{H_0,\alpha_2\gamma_2} \\ \hat{\mathcal{V}}_{H_0,\alpha_1\gamma_1} \hat{\mathcal{V}}_{H_0,\alpha_2\gamma_1} \hat{\mathcal{V}}_{H_0,\gamma_1} \hat{\mathcal{V}}_{H_0,\gamma_1\gamma_2} \\ \hat{\mathcal{V}}_{H_0,\alpha_1\gamma_2} \hat{\mathcal{V}}_{H_0,\alpha_2\gamma_2} \hat{\mathcal{V}}_{H_0,\gamma_1\gamma_2} \hat{\mathcal{V}}_{H_0,\gamma_2} \end{pmatrix}$$

Additional simulation results

Table S1: Power of the TWIST compared to the TLRT with 20% censoring

(a) α has an additive effect and $e^{\alpha} > 1$

	Underdispersion ($\gamma_1 = -0.35$ and $\gamma_2 = 0$)		Equidispersion ($\gamma_1 = 0$ and $\gamma_2 = 0$)		Overdispersion ($\gamma_1 = 0.1$ and $\gamma_2 = 0$)	
	**TWIST**	**TLRT**	**TWIST**	**TLRT**	**TWIST**	**TLRT**
**2**	0.90	0.90	0.21	0.24	0.17	0.16
**1**	0.85	0.82	0.10	0.08	0.09	0.07
**0.50**	0.90	0.89	0.16	0.15	0.10	0.10

(b) α has no effect, $e^{\alpha} = 1$

	Underdispersion ($\gamma_1 = -0.35$ and $\gamma_2 = 0$)		Equidispersion ($\gamma_1 = 0$ and $\gamma_2 = 0$)		Overdispersion ($\gamma_1 = 0.1$ and $\gamma_2 = 0$)	
	**TWIST**	**TLRT**	**TWIST**	**TLRT**	**TWIST**	**TLRT**
**2**	0.45	0.51	0.09	0.09	0.59	0.60
**1**	0.34	0.35	0.03	0.03	0.44	0.45
**0.50**	0.41	0.42	0.05	0.05	0.51	0.53

The underlined values show the estimated level of the type I error.

(c) α has an additive effect and $e^{\alpha} < 1$

	Underdispersion ($\gamma_1 = -0.35$ and $\gamma_2 = 0$)		Equidispersion ($\gamma_1 = 0$ and $\gamma_2 = 0$)		Overdispersion ($\gamma_1 = 0.1$ and $\gamma_2 = 0$)	
	**TWIST**	**TLRT**	**TWIST**	**TLRT**	**TWIST**	**TLRT**
**2**	0.11	0.16	0.33	0.27	0.93	0.91
**1**	0.06	0.07	0.20	0.14	0.92	0.87
**0.50**	0.05	0.08	0.25	0.17	0.94	0.91

α and γ both have additive effects. The tail defects for the [AA], [Aa] and [aa] groups are respectively : 0.50, 0.55 and 0.59 for γ_1 = -0.35, and 0.50, 0.44 and 0.37 for γ_1 = 0.10. The proportion of pre-immune subjects is 10% and the MAF is 20%.

Table S2: Power of the TWIST compared to the TLRT with a 10% minor allele frequency

(a) α has an additive effect and $e^{\alpha} > 1$

	**Underdispersion** ($\gamma_1 = -0.35$ and $\gamma_2 = 0$)		**Equidispersion** ($\gamma_1 = 0$ and $\gamma_2 = 0$)		**Overdispersion** ($\gamma_1 = 0.1$ and $\gamma_2 = 0$)	
	**TWIST**	**TLRT**	**TWIST**	**TLRT**	**TWIST**	**TLRT**
**2**	0.58	0.16	0.18	0.03	0.27	0.18
**1**	0.63	0.18	0.16	0.01	0.26	0.15
**0.50**	0.63	0.17	0.16	0.01	0.27	0.18

(b) α has no effect, $e^{\alpha} = 1$

	**Underdispersion** ($\gamma_1 = -0.35$ and $\gamma_2 = 0$)		**Equidispersion** ($\gamma_1 = 0$ and $\gamma_2 = 0$)		**Overdispersion** ($\gamma_1 = 0.1$ and $\gamma_2 = 0$)	
	**TWIST**	**TLRT**	**TWIST**	**TLRT**	**TWIST**	**TLRT**
**2**	0.16	0.12	0.06	0.04	0.32	0.29
**1**	0.14	0.10	0.04	0.03	0.31	0.28
**0.5**	0.19	0.13	0.06	0.04	0.33	0.31

The underlined values show the estimated level of the type I error.

(c) α has an additive effect and $e^{\alpha} < 1$

	**Underdispersion** ($\gamma_1 = -0.35$ and $\gamma_2 = 0$)		**Equidispersion** ($\gamma_1 = 0$ and $\gamma_2 = 0$)		**Overdispersion** ($\gamma_1 = 0.1$ and $\gamma_2 = 0$)	
	**TWIST**	**TLRT**	**TWIST**	**TLRT**	**TWIST**	**TLRT**
**2**	0.09	0.10	0.20	0.07	0.66	0.41
**1**	0.07	0.08	0.20	0.06	0.72	0.42
**0.50**	0.06	0.09	0.19	0.07	0.73	0.46

α and γ both have additive effects. The tail defects for the [AA], [Aa] and [aa] groups are respectively : 0.50, 0.55 and 0.59 for γ_1 = -0.35, and 0.50, 0.44 and 0.37 for $\gamma_1 = 0.10$. The proportion of pre-immune subjects is 10%.

Table S3: Power of the TWIST compared to the TLRT with a 30% minor allele frequency

(a) α has an additive effect and $e^{\alpha} > 1$

	**Underdispersion ($\gamma_1 = -0.35$ and $\gamma_2 = 0$)**		**Equidispersion ($\gamma_1 = 0$ and $\gamma_2 = 0$)**		**Overdispersion ($\gamma_1 = 0.1$ and $\gamma_2 = 0$)**	
	**TWIST**	**TLRT**	**TWIST**	**TLRT**	**TWIST**	**TLRT**
**2**	0.99	0.85	0.71	0.39	0.76	0.78
**1**	0.98	0.51	0.40	0.03	0.46	0.40
**0.5**	1	0.66	0.52	0.08	0.59	0.53

(b) α has no effect, $e^{\alpha} = 1$

	**Underdispersion ($\gamma_1 = -0.35$ and $\gamma_2 = 0$)**		**Equidispersion ($\gamma_1 = 0$ and $\gamma_2 = 0$)**		**Overdispersion ($\gamma_1 = 0.1$ and $\gamma_2 = 0$)**	
	**TWIST**	**TLRT**	**TWIST**	**TLRT**	**TWIST**	**TLRT**
**2**	0.80	0.74	0.27	0.37	0.84	0.88
**1**	0.50	0.34	<u>0.03</u>	<u>0.02</u>	0.61	0.60
**0.5**	0.63	0.50	<u>0.06</u>	<u>0.05</u>	0.75	0.77

The underlined values show the estimated level of the type I error.

(c) α has an additive effect and $e^{\alpha} < 1$

	**Underdispersion ($\gamma_1 = -0.35$ and $\gamma_2 = 0$)**		**Equidispersion ($\gamma_1 = 0$ and $\gamma_2 = 0$)**		**Overdispersion ($\gamma_1 = 0.1$ and $\gamma_2 = 0$)**	
	**TWIST**	**TLRT**	**TWIST**	**TLRT**	**TWIST**	**TLRT**
**2**	0.52	0.67	0.71	0.43	1	0.95
**1**	0.19	0.21	0.38	0.05	0.98	0.81
**0.5**	0.31	0.37	0.54	0.10	0.99	0.90

α and γ both have additive effects. The tail defects for the [AA], [Aa] and [aa] groups are respectively : 0.50, 0.55 and 0.59 for γ_1 = -0.35, and 0.50, 0.44 and 0.37 for $\gamma_1 = 0.10$. The proportion of pre-immune subjects is 10%.

Table S4: Power of the TWIST compared to the TLRT with 5% pre-immune subjects

(a) α has an additive effect and $e^{\alpha} > 1$

	**Underdispersion** ($\gamma_1 = -0.35$ and $\gamma_2 = 0$)		**Equidispersion** ($\gamma_1 = 0$ and $\gamma_2 = 0$)		**Overdispersion** ($\gamma_1 = 0.1$ and $\gamma_2 = 0$)	
	**TWIST**	**TLRT**	**TWIST**	**TLRT**	**TWIST**	**TLRT**
**2**	0.95	0.50	0.39	0.06	0.44	0.35
**1**	0.93	0.38	0.32	0.03	0.40	0.30
**0.5**	0.96	0.44	0.31	0.02	0.41	0.32

(b) α has no effect, $e^{\alpha} = 1$

	**Underdispersion** ($\gamma_1 = -0.35$ and $\gamma_2 = 0$)		**Equidispersion** ($\gamma_1 = 0$ and $\gamma_2 = 0$)		**Overdispersion** ($\gamma_1 = 0.1$ and $\gamma_2 = 0$)	
	**TWIST**	**TLRT**	**TWIST**	**TLRT**	**TWIST**	**TLRT**
**2**	0.50	0.38	0.06	0.05	0.56	0.56
**1**	0.40	0.28	<u>0.03</u>	<u>0.03</u>	0.50	0.49
**0.5**	0.44	0.32	<u>0.03</u>	<u>0.03</u>	0.50	0.49

The underlined values show the estimated level of the type I error.

(c) α has an additive effect and $e^{\alpha} < 1$

	**Underdispersion** ($\gamma_1 = -0.35$ and $\gamma_2 = 0$)		**Equidispersion** ($\gamma_1 = 0$ and $\gamma_2 = 0$)		**Overdispersion** ($\gamma_1 = 0.1$ and $\gamma_2 = 0$)	
	**TWIST**	**TLRT**	**TWIST**	**TLRT**	**TWIST**	**TLRT**
**2**	0.20	0.27	0.38	0.10	0.95	0.77
**1**	0.13	0.16	0.33	0.06	0.94	0.67
**0.5**	0.16	0.20	0.35	0.06	0.94	0.70

α and γ both have additive effects. The tail defects for the [AA], [Aa] and [aa] groups are respectively : 0.50, 0.55 and 0.59 for γ_1 = -0.35, and 0.50, 0.44 and 0.37 for γ_1 = 0.10. The MAF is 20%.

Table S5: Power of the TWIST compared to the TLRT with 20% pre-immune subjects

(a) α has an additive effect and $e^{\alpha} > 1$

	**Underdispersion** ($\gamma_1 = -0.35$ and $\gamma_2 = 0$)		**Equidispersion** ($\gamma_1 = 0$ and $\gamma_2 = 0$)		**Overdispersion** ($\gamma_1 = 0.1$ and $\gamma_2 = 0$)	
	**TWIST**	**TLRT**	**TWIST**	**TLRT**	**TWIST**	**TLRT**
**2**	0.93	0.57	0.43	0.15	0.51	0.47
**1**	0.89	0.32	0.25	0.03	0.32	0.23
**0.5**	0.94	0.51	0.37	0.06	0.44	0.39

(b) α has no effect, $e^{\alpha} = 1$

	**Underdispersion** ($\gamma_1 = -0.35$ and $\gamma_2 = 0$)		**Equidispersion** ($\gamma_1 = 0$ and $\gamma_2 = 0$)		**Overdispersion** ($\gamma_1 = 0.1$ and $\gamma_2 = 0$)	
	**TWIST**	**TLRT**	**TWIST**	**TLRT**	**TWIST**	**TLRT**
**2**	0.50	0.48	0.17	0.17	0.61	0.62
**1**	0.31	0.21	<u>0.03</u>	<u>0.02</u>	0.41	0.41
**0.5**	0.47	0.38	<u>0.06</u>	<u>0.06</u>	0.58	0.57

The underlined values show the estimated level of the type I error.

(c) α has an additive effect and $e^{\alpha} < 1$

	**Underdispersion** ($\gamma_1 = -0.35$ and $\gamma_2 = 0$)		**Equidispersion** ($\gamma_1 = 0$ and $\gamma_2 = 0$)		**Overdispersion** ($\gamma_1 = 0.1$ and $\gamma_2 = 0$)	
	**TWIST**	**TLRT**	**TWIST**	**TLRT**	**TWIST**	**TLRT**
**2**	0.26	0.39	0.45	0.22	0.92	0.75
**1**	0.10	0.11	0.26	0.05	0.89	0.60
**0.5**	0.19	0.24	0.42	0.11	0.95	0.75

α and γ both have additive effects. The tail defects for the [AA], [Aa] and [aa] groups are respectively : 0.50, 0.55 and 0.59 for γ_1 = -0.35, and 0.50, 0.44 and 0.37 for γ_1 = 0.10. The MAF is 20%.

Table S6: Power of the TWIST compared to the TLRT with a 30% tail defect for the reference group

(a) α has an additive effect and $e^{\alpha} > 1$

	**Underdispersion ($\gamma_1 = -0.60$ and $\gamma_2 = 0$)**		**Equidispersion ($\gamma_1 = 0$ and $\gamma_2 = 0$)**		**Overdispersion ($\gamma_1 = 0.17$ and $\gamma_2 = 0$)**	
	**TWIST**	**TLRT**	**TWIST**	**TLRT**	**TWIST**	**TLRT**
**2**	0.99	0.77	0.50	0.10	0.51	0.48
**1**	0.98	0.66	0.37	0.04	0.40	0.33
**0.50**	0.99	0.75	0.45	0.07	0.48	0.41

(b) α has no effect, $e^{\alpha} = 1$

	**Underdispersion ($\gamma_1 = -0.60$ and $\gamma_2 = 0$)**		**Equidispersion ($\gamma_1 = 0$ and $\gamma_2 = 0$)**		**Overdispersion ($\gamma_1 = 0.17$ and $\gamma_2 = 0$)**	
	**TWIST**	**TLRT**	**TWIST**	**TLRT**	**TWIST**	**TLRT**
**2**	0.62	0.54	0.09	0.10	0.78	0.81
**1**	0.48	0.37	0.03	0.02	0.69	0.67
**0.50**	0.58	0.46	0.04	0.03	0.76	0.73

The underlined values show the estimated level of the type I error.

(c) α has an additive effect and $e^{\alpha} < 1$

	**Underdispersion ($\gamma_1 = -0.60$ and $\gamma_2 = 0$)**		**Equidispersion ($\gamma_1 = 0$ and $\gamma_2 = 0$)**		**Overdispersion ($\gamma_1 = 0.17$ and $\gamma_2 = 0$)**	
	**TWIST**	**TLRT**	**TWIST**	**TLRT**	**TWIST**	**TLRT**
**2**	0.24	0.31	0.50	0.19	1	0.95
**1**	0.14	0.17	0.39	0.08	0.99	0.91
**0.50**	0.20	0.22	0.49	0.12	1	0.94

α and γ both have additive effects. The tail defects for the [AA], [Aa] and [aa] groups are respectively : 0.30, 0.39 and 0.45 for γ_1 = -0.60, and 0.30, 0.20 and 0.07 for $\gamma_1 = 0.17$. The proportion of pre-immune subjects is 10% and the MAF is 20%.

Table S7: Power of the TWIST compared to the TLRT under different genetic models

(a) $e^{\alpha} > 1$

		Dominant γ				Overdominant γ				Additive γ				Recessive γ			
		Underdispersion		Overdispersion		Underdispersion		Overdispersion		Underdispersion		Overdispersion		Underdispersion		Overdispersion	
		$\gamma_1 = -0.35$ and $\gamma_2 = -0.35$		$\gamma_1 = 0.10$ and $\gamma_2 = 0.10$		$\gamma_1 = 0$ and $\gamma_2 = -0.70$		$\gamma_1 = 0$ and $\gamma_2 = 0.20$		$\gamma_1 = -0.35$ and $\gamma_2 = 0$		$\gamma_1 = 0.10$ and $\gamma_2 = 0$		$\gamma_1 = -0.35$ and $\gamma_2 = 0.35$		$\gamma_1 = 0.10$ and $\gamma_2 = -0.10$	
e^{β}	α	TWIST	TLRT	TWIST	TLRT	TWIST	TLRT	TWIST	TLRT	TWIST	TLRT	TWIST	TLRT	TWIST	TLRT	TWIST	TLRT
**2**	**overdominant**	1	0.91	0.89	0.87	1	0.90	0.94	0.91	1	0.63	0.85	0.48	0.87	0.21	0.94	0.48
**2**	**dominant**	1	0.92	0.94	0.90	1	0.91	0.92	0.90	0.99	0.61	0.79	0.34	0.94	0.24	0.91	0.36
**2**	**additive**	0.99	0.84	0.93	0.94	1	0.87	0.93	0.95	0.95	0.56	0.49	0.42	0.60	0.21	0.48	0.29
**2**	**recessive**	0.91	0.77	0.99	0.98	0.96	0.81	0.99	0.98	0.72	0.46	0.55	0.54	0.44	0.19	0.32	0.30
**1**	**overdominant**	1	0.85	0.84	0.80	1	0.83	0.91	0.85	1	0.47	0.80	0.34	0.80	0.09	0.91	0.37
**1**	**dominant**	1	0.82	0.87	0.79	1	0.86	0.87	0.81	1	0.47	0.67	0.22	0.88	0.11	0.84	0.24
**1**	**additive**	0.99	0.73	0.88	0.88	1	0.78	0.87	0.89	0.93	0.36	0.37	0.28	0.49	0.08	0.42	0.22
**1**	**recessive**	0.86	0.63	0.98	0.95	0.92	0.68	0.97	0.96	0.62	0.29	0.40	0.35	0.34	0.09	0.24	0.17
**0.5**	**overdominant**	1	0.88	0.87	0.82	1	0.88	0.93	0.88	1	0.53	0.84	0.42	0.84	0.14	0.95	0.43
**0.5**	**dominant**	1	0.88	0.92	0.86	1	0.90	0.92	0.88	1	0.56	0.76	0.30	0.94	0.16	0.89	0.30
**0.5**	**additive**	1	0.81	0.93	0.94	1	0.84	0.89	0.93	0.95	0.47	0.42	0.32	0.62	0.16	0.49	0.25
**0.5**	**recessive**	0.91	0.73	0.99	0.97	0.98	0.77	0.98	0.98	0.71	0.38	0.51	0.44	0.44	0.12	0.28	0.21

When α is dominant, $\alpha_1 = \alpha_2 = 0.5 \times log(2)$. When α is overdominant, $\alpha_1 = 0$ and $\alpha_2 = log(2)$. When α is additive, $\alpha_1 = 0.5 \times log(2)$ and $\alpha_2 = 0$. When α is recessive, $\alpha_1 = 0.5 \times log(2)$ and $\alpha_2 = -0.5 \times log(2)$.

(b) $e^{\alpha} < 1$

		Dominant γ				Overdominant γ				Additive γ				Recessive γ			
		Underdispersion		Overdispersion		Underdispersion		Overdispersion		Underdispersion		Overdispersion		Underdispersion		Overdispersion	
		$\gamma_1 = -0.35$ and $\gamma_2 = -0.35$		$\gamma_1 = 0.10$ and $\gamma_2 = 0.10$		$\gamma_1 = 0$ and $\gamma_2 = -0.70$		$\gamma_1 = 0$ and $\gamma_2 = 0.20$		$\gamma_1 = -0.35$ and $\gamma_2 = 0$		$\gamma_1 = 0.10$ and $\gamma_2 = 0$		$\gamma_1 = -0.35$ and $\gamma_2 = 0.35$		$\gamma_1 = 0.10$ and $\gamma_2 = -0.10$	
e^{β}	α	TWIST	TLRT	TWIST	TLRT	TWIST	TLRT	TWIST	TLRT	TWIST	TLRT	TWIST	TLRT	TWIST	TLRT	TWIST	TLRT
**2**	**overdominant**	0.55	0.60	1	1	0.64	0.64	1	1	0.59	0.29	0.99	0.80	0.92	0.25	0.91	0.46
**2**	**dominant**	0.65	0.62	1	1	0.53	0.61	1	1	0.49	0.26	1	0.84	0.87	0.20	0.97	0.54
**2**	**additive**	0.75	0.75	1	0.99	0.62	0.70	1	1	0.22	0.32	0.95	0.77	0.34	0.16	0.80	0.52
**2**	**recessive**	0.93	0.79	0.98	0.98	0.91	0.79	0.99	0.98	0.45	0.39	0.80	0.67	0.11	0.14	0.70	0.51
**1**	**overdominant**	0.39	0.40	1	0.99	0.49	0.45	1	1	0.46	0.12	0.99	0.67	0.86	0.12	0.87	0.28
**1**	**dominant**	0.56	0.47	1	1	0.41	0.44	1	1	0.33	0.12	0.99	0.72	0.78	0.10	0.95	0.44
**1**	**additive**	0.66	0.58	1	0.98	0.51	0.55	1	0.99	0.11	0.14	0.91	0.65	0.21	0.06	0.75	0.42
**1**	**recessive**	0.94	0.69	0.97	0.95	0.86	0.69	0.99	0.97	0.31	0.23	0.80	0.59	0.04	0.05	0.73	0.43
**0.5**	**overdominant**	0.49	0.51	1	1	0.62	0.58	1	1	0.55	0.15	1	0.74	0.90	0.18	0.91	0.38
**0.5**	**dominant**	0.65	0.55	1	1	0.48	0.52	1	1	0.40	0.15	1	0.79	0.84	0.13	0.96	0.54
**0.5**	**additive**	0.73	0.67	1	0.98	0.58	0.63	1	0.99	0.14	0.18	0.96	0.74	0.27	0.11	0.84	0.53
**0.5**	**recessive**	0.95	0.74	0.98	0.97	0.89	0.74	1	0.99	0.38	0.29	0.84	0.65	0.06	0.08	0.76	0.48

When α is dominant, $\alpha_1 = \alpha_2 = 0.5 \times log(0.5)$. When α is overdominant, $\alpha_1 = 0$ and $\alpha_2 = log(0.5)$. When α is additive, $\alpha_1 = 0.5 \times log(0.5)$ and $\alpha_2 = 0$. When α is recessive, $\alpha_1 = 0.5 \times log(0.5)$ and $\alpha_2 = -0.5 \times log(0.5)$.

(c) Power of the TWIST and the TLRT with various genetic models for γ and $\alpha = 0$

	Overdominant γ				Dominant γ				Additive γ				Recessive γ			
	Underdispersion		Overdispersion		Underdispersion		Overdispersion		Underdispersion		Overdispersion		Underdispersion		Overdispersion	
	$\gamma_1 = 0$ and $\gamma_2 = -0.7$		$\gamma_1 = 0$ and $\gamma_2 = 0.2$		$\gamma_1 = -0.35$ and $\gamma_2 = -0.35$		$\gamma_1 = 0.1$ and $\gamma_2 = 0.1$		$\gamma_1 = -0.35$ and $\gamma_2 = 0$		$\gamma_1 = 0.1$ and $\gamma_2 = 0$		$\gamma_1 = -0.35$ and $\gamma_2 = 0.35$		$\gamma_1 = 0.1$ and $\gamma_2 = -0.1$	
e^{β}	TWIST	TLRT	TWIST	TLRT	TWIST	TLRT	TWIST	TLRT	TWIST	TLRT	TWIST	TLRT	TWIST	TLRT	TWIST	TLRT
**2**	0.91	0.77	0.98	0.98	0.91	0.81	0.99	0.99	0.50	0.44	0.59	0.60	0.17	0.18	0.40	0.41
**1**	0.81	0.64	0.96	0.94	0.85	0.65	0.98	0.96	0.36	0.25	0.50	0.48	0.09	0.07	0.30	0.30
**0.50**	0.88	0.73	0.97	0.96	0.91	0.75	0.99	0.98	0.45	0.34	0.53	0.53	0.13	0.12	0.38	0.37

$\alpha_1 = \alpha_2 = 0.$

The proportion of pre-immune subjects is 10% and the MAF is 20%.

Table S8: Power of the TWIST and the TLRT with $\gamma = 0$ and various genetic models for α.

(a) $e^{\alpha} > 1$

		Equidispersion ($\gamma_1 = 0$ and $\gamma_2 = 0$)	
e^{β}	α	TWIST	TLRT
2	overdominant	0.84	0.12
2	dominant	0.85	0.11
2	additive	0.42	0.08
2	recessive	0.20	0.09
1	overdominant	0.76	0.05
1	dominant	0.77	0.03
1	additive	0.29	0.02
1	recessive	0.11	0.02
0.5	overdominant	0.81	0.07
0.5	dominant	0.84	0.06
0.5	additive	0.32	0.03
0.5	recessive	0.15	0.03

When α is dominant, $\alpha_1 = \alpha_2 = 0.5 \times log(2)$. When α is overdominant, $\alpha_1 = 0$ and $\alpha_2 = log(2)$. When α is additive, $\alpha_1 = 0.5 \times log(2)$ and $\alpha_2 = 0$. When α is recessive, $\alpha_1 = 0.5 \times log(2)$ and $\alpha_2 = -0.5 \times log(2)$.

(b) $e^{\alpha} < 1$

		Equidispersion ($\gamma_1 = 0$ and $\gamma_2 = 0$)	
e^{β}	α	TWIST	TLRT
2	overdominant	0.83	0.15
2	dominant	0.87	0.16
2	additive	0.41	0.12
2	recessive	0.22	0.11
1	overdominant	0.78	0.05
1	dominant	0.79	0.06
1	additive	0.30	0.04
1	recessive	0.14	0.04
0.5	overdominant	0.83	0.07
0.5	dominant	0.85	0.08
0.5	additive	0.35	0.05
0.5	recessive	0.17	0.06

When α is dominant, $\alpha_1 = \alpha_2 = 0.5 \times log(0.5)$. When α is overdominant, $\alpha_1 = 0$ and $\alpha_2 = log(0.5)$. When α is additive, $\alpha_1 = 0.5 \times log(0.5)$ and $\alpha_2 = 0$. When α is recessive, $\alpha_1 = 0.5 \times log(0.5)$ and $\alpha_2 = -0.5 \times log(0.5)$.

The proportion of pre-immune subjects is 10% and the MAF is 20%.
